# Study of Nanoscale Friction Behaviors of Graphene on Gold Substrates Using Molecular Dynamics

**DOI:** 10.1186/s11671-018-2451-3

**Published:** 2018-02-02

**Authors:** Pengzhe Zhu, Rui Li

**Affiliations:** 1 School of Mechanical, Electronic and Control Engineering, Beijing Jiaotong University, Beijing, 100044 China; 2 Key Laboratory of Vehicle Advanced Manufacturing, Measuring and Control Technology, Ministry of Education, Beijing, 100044 China; 3 School of Mechanical Engineering, University of Science and Technology Beijing, Beijing, 100083 China

**Keywords:** Graphene, Friction, Single crystalline gold, Molecular dynamics

## Abstract

In this paper, we investigate the friction behaviors of graphene flakes sliding on a gold substrate using molecular dynamics simulations. The effects of flake size, flake shape, relative rotation angle between flake and substrate, and crystal orientation of substrate on the friction process are thoroughly studied. It is found that under the same load, the average friction forces per atom are smaller for a bigger graphene flake, which exhibits an obvious size effect. It is also shown that flake shape is critical in determining the friction in the sliding process. The average friction forces per atom for the square flake are much bigger than those for the triangular and round flakes. Moreover, the average friction forces per atom for the triangular flake are the smallest. We also find that the orientation of graphene flake relative to gold substrate plays a vital role in the friction process. The friction forces for the graphene flake sliding along the armchair direction are much bigger than those for the flakes with rotation. In addition, it is also found that single crystalline gold substrate exhibits a significant anisotropic effect of friction, which is attributed to the anisotropic effect of potential energy corrugation. These understandings not only shed light on the underlying mechanisms of graphene flake sliding on the gold substrates but also may guide the design and fabrication of nanoscale graphene-based devices.

## Background

Graphene is one of the promising new materials for application in nanoscale electronics among a wide range of potential applications [1–5]. In actual graphene-based electronic devices, gold is commonly used for electric contacts [6]. Therefore, the friction of graphene-gold system plays an important role in the efficient fabrication and reliable operation of such graphene devices. Although graphene has attracted great interest from the researchers in the field of nanotribology due to its excellent mechanical properties [3, 7], the friction properties of graphene sliding on gold surface are poorly understood. So far, many tribological studies of graphene focus on the friction force between graphene and a scanning probe tip [8–14]. For instance, the atomic force microscopy (AFM) experiments of friction on chemically modified graphite revealed a negative friction coefficient [9]. The friction force microscope (FFM) experiments of few-layer graphene found that friction increased as the number of graphene layers decreased [10, 11]. These phenomena are explained by the puckering effect of graphene [9–11]. It is supposed that the friction between a graphene flake and graphite was measured as the tip dragged a flake during sliding on graphite [8, 14]. It is found that the rotational motion coupled to lateral motion of flakes for lamellar solids leads to friction increase due to flake reorientation into a commensurate configuration [15]. At the same time, some scholars have also been devoted to studying the friction of graphene and/or gold using other techniques. Quartz crystal microbalance (QCM) technique was employed to study the lubricity of gold on graphene [16] and the sliding friction of solid xenon film on graphene/Ni(111) substrate [17]. Both QCM experiments and molecular dynamics (MD) simulations show that the friction of an incommensurate Kr monolayer on Au follows a viscous friction law [18, 19]. MD simulations are conducted to explore the static friction of two-dimensional gold islands and three-dimensional gold clusters on graphite substrate [20]. It is found that the slider thickness can promote lubricity due to the higher effective rigidity of thick clusters. The size-dependent interfacial commensurability was also uncovered by MD simulations of xenon atoms on graphene and Au substrate [21], consistent with the simulations of krypton and silicon clusters on Cu substrate [22], which can explain the size dependence of static friction. Recently, the superlubricity of graphene nanoribbons on an Au(111) substrate is observed at a low temperature [23]. Kitt et al. directly measured the friction of graphene sliding over a SiO_2_ substrate and found that the friction behaviors for monolayer and bilayer graphene violate Amontons’ law [24]. Overall, as a pure two-dimensional material, it is reasonable to expect graphene to exhibit atypical friction behaviors for graphene-substrate system. Unfortunately, a detailed investigation of sliding friction of graphene over a gold substrate is still lacking although the interfacial properties between graphene and metals have been systematically explored [25–28].

To fill this gap, in this paper, the sliding friction behaviors of mobile graphene flakes over a single crystalline gold substrate are thoroughly studied using molecular dynamics (MD) simulations. We investigate the effects of flake size, flake shape, relative rotation angle between flake and substrate, and crystal orientation of substrate to clarify the friction properties.

## Methods

### Simulation Method

**Fig. 1:**
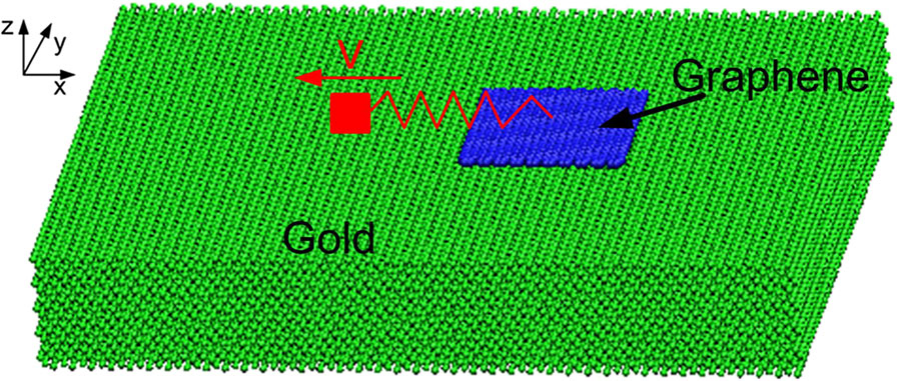
Simulation model of friction process

To simulate an AFM experiment where a graphene flake attached through a spring to a tip slides over a gold substrate [29], we establish a MD model consisting of a graphene flake made of *N* atoms and a single crystalline gold substrate, see Fig. 1. The three layers of atoms at the bottom of the substrate are kept fixed in space to serve as boundary atoms. To control the temperature of the system, four layers of atoms adjacent to the boundary atoms in the substrate are chosen as thermostat atoms. The thermostat atoms are kept at a constant temperature of 300 K by the velocity scaling method [30]. In this paper, first, we do not consider the rotation of graphene during sliding; the atoms in the graphene are only allowed to move in the *x* and *z* directions but are constrained in the *y* direction, which simplifies the simulations. This is the main focus of this paper. Then, we further perform some MD simulations without the movement constraint of graphene in the *y* direction to better match the real experimental conditions. The flake atoms are dragged by a virtual atom with a constant velocity through a harmonic spring. The spring parallel to substrate surface has a lateral stiffness of 10 N/m and is used to represent the deformation of the cantilever beam and tip apex of an AFM system [31]. A constant normal load is applied directly to the flake atoms in the simulations [29, 31]. The graphene flake is pulled laterally by a virtual atom at a constant speed of 10 m/s. The equations of motion are integrated with a velocity-Verlet algorithm. The timestep is 1 fs. Boundaries are periodic in the *x* and *y* directions, free in the *z* direction.

To investigate the effect of crystal orientation of substrate, we perform MD simulations of sliding friction on three different surfaces of Au(111), (001), and (110) planes, respectively. For Au(111) surface, the coordinate systems are taken as $x-\left[11\overline{2}\right]$, $y-\left[1\overline{1}0\right]$_,_ and *z*-[111] and the size is 19.98 × 15.0 × 3.06 nm^3^. For Au(001) surface, the coordinate systems are taken as *x*-[100], *y*-[010], and *z-*[001] and the size of substrate is 19.99 × 15.1 × 3.06 nm^3^. For Au(110) surface, the coordinate systems are taken as *x*-[001], $y-\left[1\overline{1}0\right]$_,_ and *z*-[110] and the size is 19.99 × 15.0 × 3.03 nm^3^. The lattice spacings along the sliding direction for Au(111), Au(110), and Au(001) surfaces are 9.99 Å, 4.08 Å, and 4.08 Å, respectively. If not noted, the Au(111) is adopted as substrate. In the simulations, several different flake sizes and shapes are modeled. The default flake shape is a square with a size of 5.8 nm (the number of atoms *N* = 1344). The *x*-axis is along the armchair direction of the graphene and the *y*-axis is along the zigzag direction, with the *z*-axis normal to the graphene.

Interatomic forces within Au are derived from an embedded atom method (EAM) potential [32]. The EAM potential has been very successful in modeling the elastic properties, defect formation energies, and fracture mechanisms of various metals [32, 33]. It has also been successfully applied to describe the surface properties of metals such as surface energies and surface reconstructions [32–34]. The widely used AIREBO potential is applied to describe the interaction of atoms within graphene [35]. The interaction between graphene and Au substrate is modeled by the standard Lennard-Jones (LJ) potential which has been employed to study many non-equilibrium phenomena such as friction and diffusion of gold clusters on graphite [36, 37]. The LJ parameters [28, 29] are: *ε* = 22.0 meV and *σ* = 2.74 Å. The MD simulations are conducted using the large-scale atomic/molecular massively parallel simulator (LAMMPS) [38]. In the simulations, the graphene flake is initially positioned above the Au substrate surface. After the friction system is fully relaxed, the virtual atom starts sliding along the negative *x* direction with a constant velocity.

## Results and Discussions

**Fig. 2:**
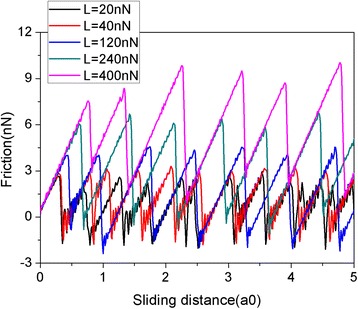
Friction force as a function of sliding distance at various normal loads (L). The flake has a square shape with a size of 5.8 nm. Here, a0 (= 9.99 Å) is the lattice spacing of Au(111) along the sliding direction

**Fig. 3:**
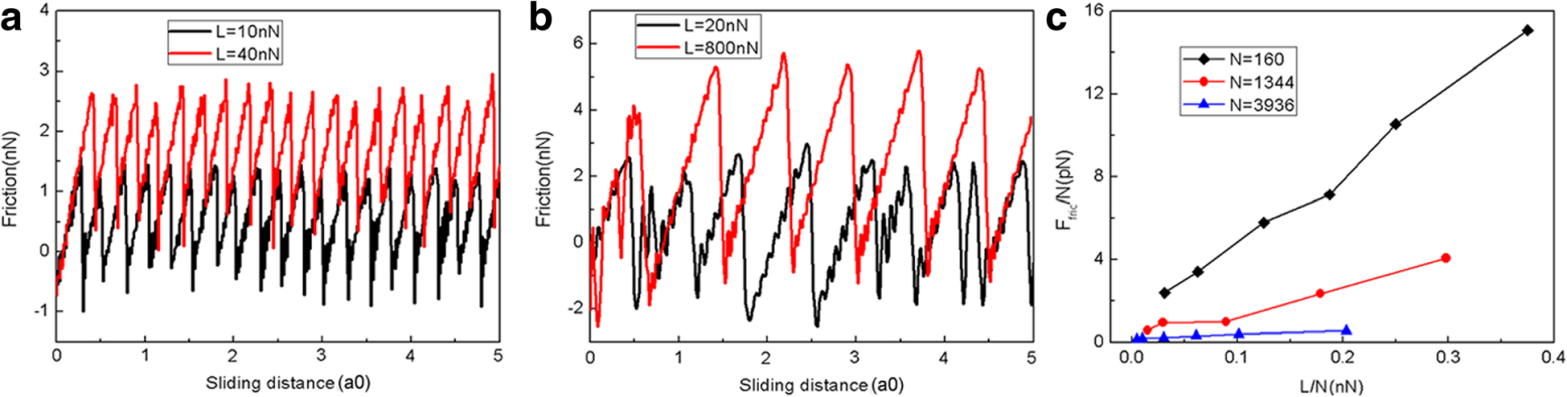
The variation of friction force and average friction force for different flake sizes. The typical friction force as a function of sliding distance for the 2.0 nm (*N* = 160 atoms) flake (**a**) and 10 nm (*N* = 3936 atoms) flake (**b**). **c** The average friction force per atom (*F*_fric_/N) as a function of load per atom (L/N). Here, a0 (= 9.99 Å) is the lattice spacing of Au(111) along the sliding direction

Figure 2 shows the friction force as a function of sliding distance at various normal loads. In this paper, the sliding distance is that of the virtual atom. The friction force is measured by the deformation of the spring as in an AFM experiment. The graphene flake has a square shape with a size of 5.8 nm consisting of 1344 atoms. It is clear that the friction forces experience continuous increase followed by sharp drops, which is typical of stick-slip motion. The abrupt drops in the friction force lead to energy dissipation and imply the occurrence of transitions between multiple metastable states with local potential energy minima [39]. It is reasonable that the friction force increases with the load L. To explore the size effect, another two square flakes with sizes of 2.0 nm (*N* = 160 atoms) and 10.0 nm (*N* = 3936 atoms) are adopted. The variation of friction force and average friction force for different flake sizes during the sliding process are shown in Fig. 3. As for the 5.8-nm flake, an obvious stick-slip friction can also be observed for both the 2.0- and 10-nm flakes. Moreover, there clearly exists a size effect in the average friction force per atom *F*_fric_/N, see Fig. 3c. Under the same load, the average friction forces per atom *F*_fric_/N are bigger for a smaller flake. This size effect results from the progressively decreasing role of edges in the friction with the increasing flake size [40, 41]. It should be noted that both the QCM experiments and MD simulations found that the friction of adsorbate clusters decreases with the increase of their size [18–22], in agreement with our simulations. However, the size dependence of friction in the QCM experiments and MD simulations is explained by the size-dependent interfacial commensurability [18–22].

**Fig. 4:**
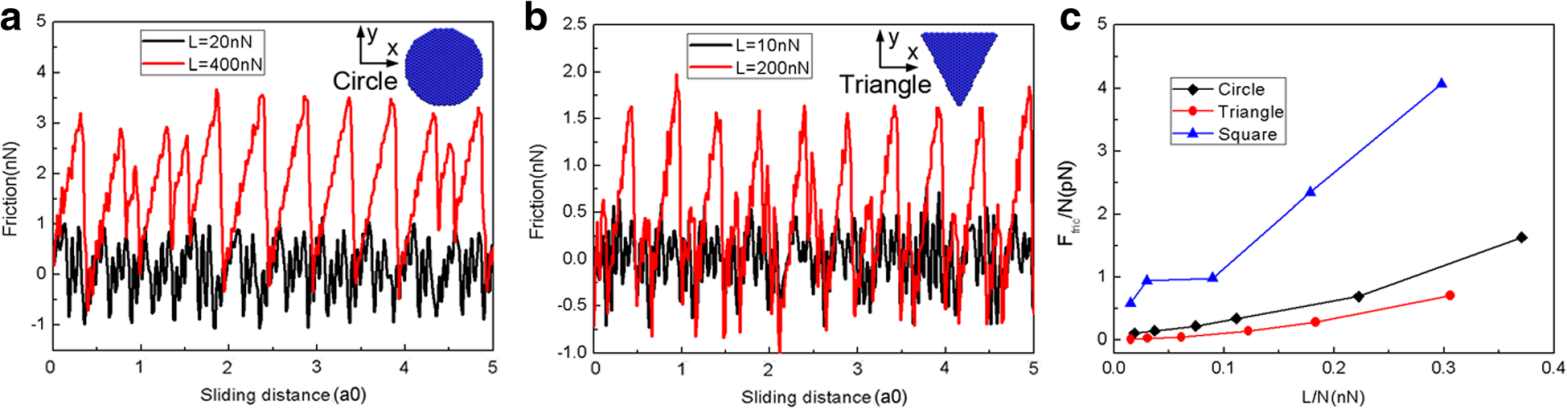
The variation of friction force and average friction force for different flake shapes. The typical friction force as a function of sliding distance for the round (*N* = 1080 atoms) flake (**a**) and triangular (*N* = 654 atoms) flake (**b**). **c** The average friction force per atom (*F*_fric_/N) as a function of load per atom (L/N). Here, a0 (= 9.99 Å) is the lattice spacing of Au(111) along the sliding direction

As the slider shape plays a significant role in determining friction [42, 43], to further explore the effect of flake shape on the friction process, we also model the sliding friction process using a round graphene flake (*N* = 1080 atoms) and a triangular graphene flake (*N* = 654 atoms). Figure 4 shows the variation of the typical friction forces and average friction forces for different flake shapes during the sliding process. As shown in Fig. 4a, b, at small loads (*L* = 20 nN for round flake and *L* = 10 nN for triangular flake), the friction force fluctuates around zero continuously and super low friction (superlubricity) can be observed. However, at large normal loads (*L* = 400 nN for round flake and *L* = 200 nN for triangular flake), the flake exhibits obvious stick-slip motion and a large friction force [39]. Under the same load, the average friction forces per atom *F*_fric_/N are the biggest for the square flake and the smallest for the triangular flake, whereas *F*_fric_/N for the round flake are in between. Furthermore, the difference of the average friction forces per atom *F*_fric_/N between the round and triangular flake is rather small. But *F*_fric_/N for the square flake is much bigger. Therefore, it is clear that the flake shape plays a vital role in the sliding process.

**Fig. 5:**
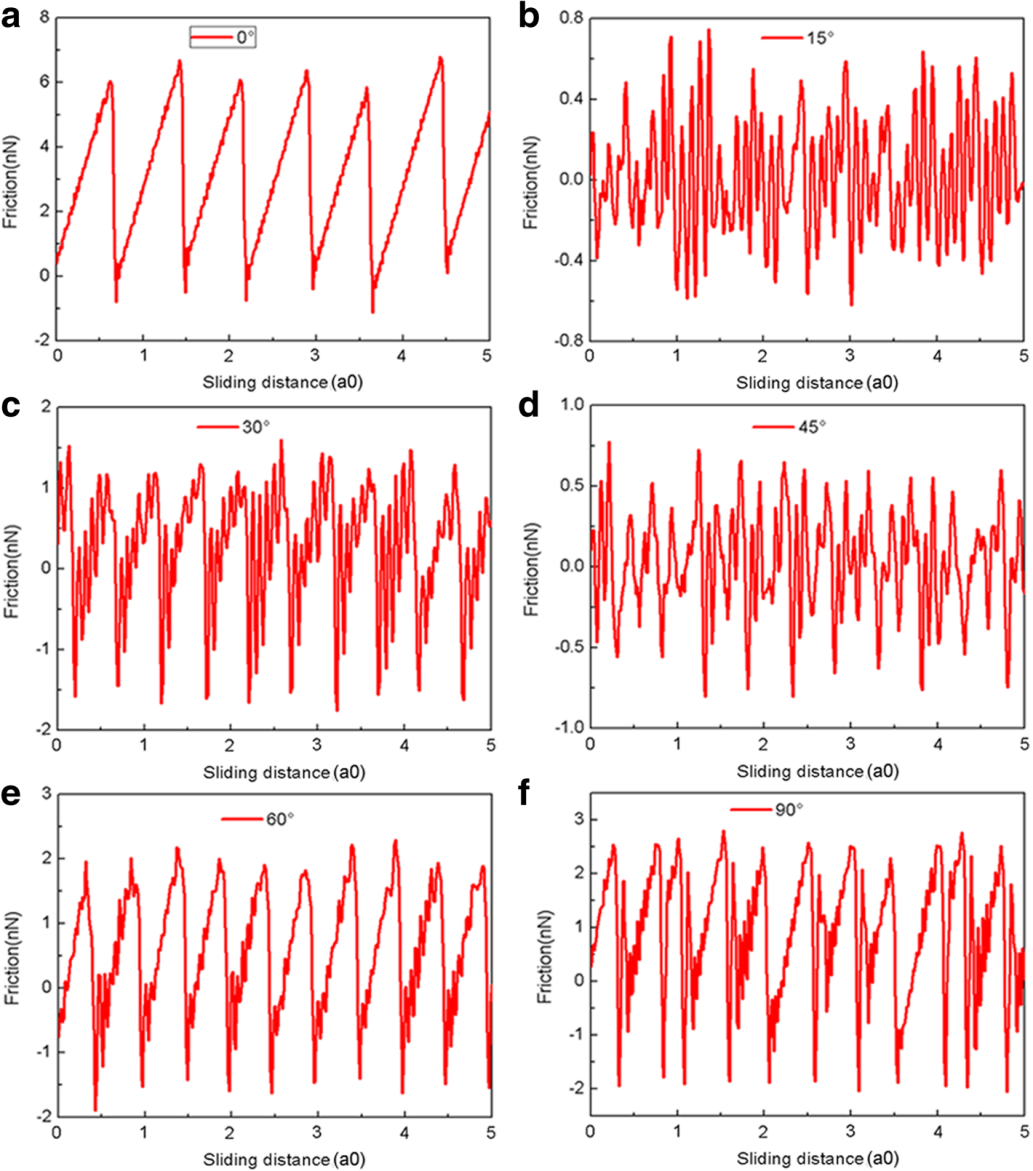
The friction force vs sliding distance of the 5.8 nm square flake at *L* = 240 nN for different rotation angles (θ = 0°, 15°, 30°, 45°, 60°, 90°). **a**–**f** correspond to the rotation angle 0°~90°, respectively. Here, a0 (= 9.99 Å) is the lattice spacing of Au(111) along the sliding direction

**Fig. 6:**
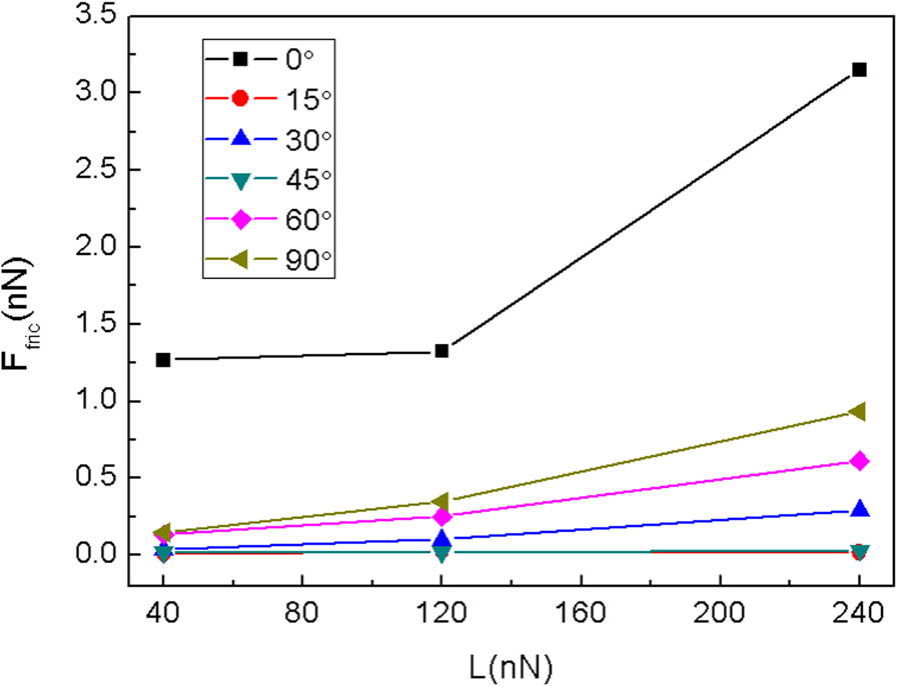
The average friction force Ffric of the 5.8-nm square flake for different rotation angles at different normal loads

It is well-known that the orientation of flake relative to the substrate is also critical in determining the friction [42]. To explore the orientation effect on the friction, the graphene flake is rotated anticlockwise by different angles about the *z*-axis perpendicular to the contact. The rotation angle 0° (without rotation) corresponds to the situation where the *x*-axis is along the armchair direction of the graphene while the rotation angle 90° corresponds to the situation where the *x*-axis is along the zigzag direction. The variation of friction force as a function of sliding distance for the 5.8-nm square flake with different rotation angles at *L* = 240 nN is shown in Fig. 5. The corresponding average friction forces *F*_fric_ for different rotation angles at different normal loads are calculated as shown in Fig. 6. It is obvious that for flakes with θ = 15° and θ = 45°, the friction forces fluctuate around zero continuously and superlubricity can be observed, see Fig. 5b, d. Moreover, little difference can be observed in the average friction forces for flakes with θ = 15° and θ = 45°, see Fig. 6. However, for the flakes with θ = 30°, 60° and θ = 90°, the flakes exhibit obvious stick-slip motion and a relatively large friction force. Furthermore, the average friction force is bigger for a larger rotation angle for the flakes with θ = 30°, 60°, and θ = 90°. The friction forces for flakes with rotation are all much smaller than that for the flake without rotation (θ = 0°).

**Fig. 7:**
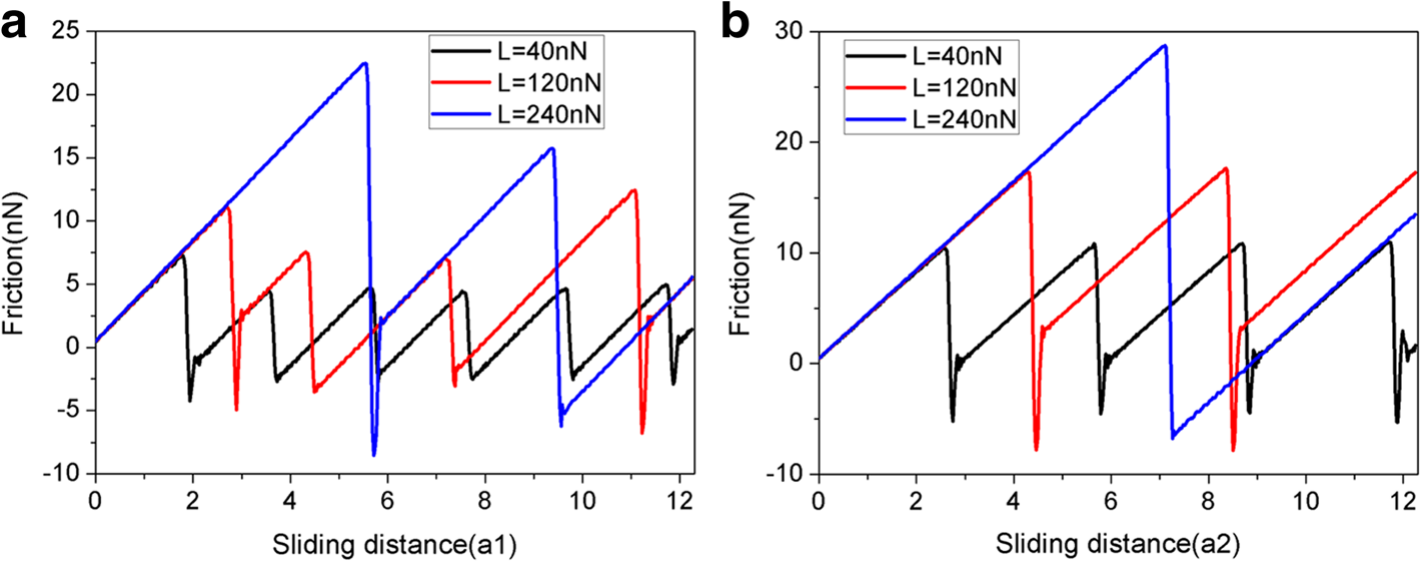
The friction force of the 5.8-nm square flake sliding on the (**a**) Au(001) and (**b**) Au(110) surfaces as a function of sliding distance at different normal loads. Here, a1 (= 4.08 Å) is the lattice spacing of Au(001) along the sliding direction and a2 (= 4.08 Å) is lattice spacing of Au(110) along the sliding direction

**Fig. 8:**
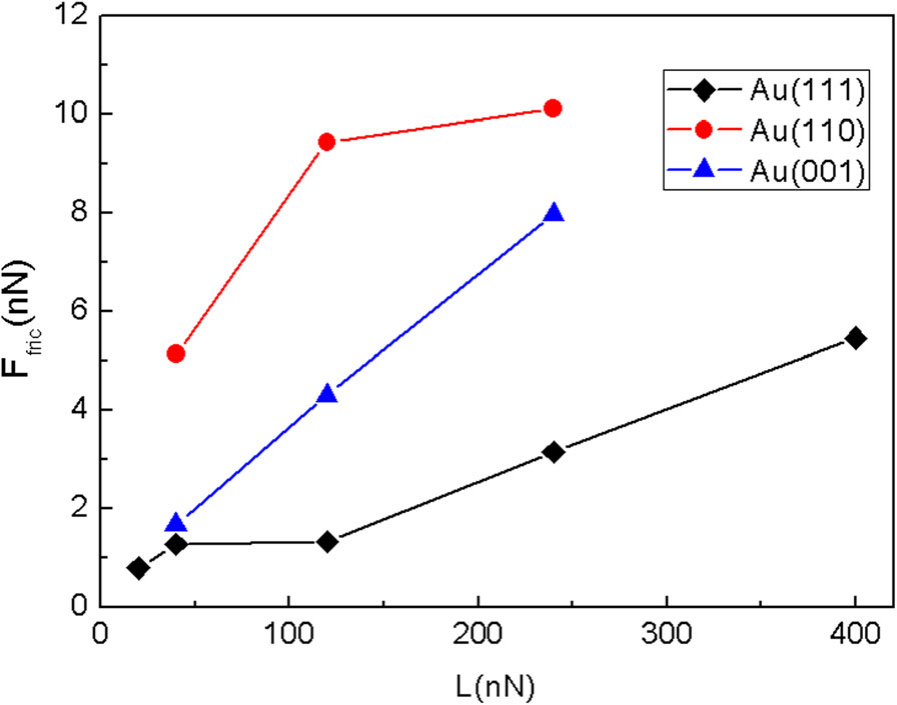
The average friction force *F*_fric_ of the 5.8-nm square flake sliding on the Au substrates with different crystal orientation at different normal loads

The fact that single crystalline gold exhibits significant anisotropic effects encourages us to further study the effect of crystal orientation of substrate on the friction process. We carried out MD simulations for two more combinations of crystal orientation and sliding direction, i.e., (001) [100] and (110) [001]. The friction force and average friction force *F*_fric_ of the 5.8-nm square flake sliding on the Au substrates with different crystal orientation are shown in Figs. 7 and 8, respectively. As expected, the friction force increases with the normal load. It can be seen that under the same load, the friction forces for the Au(001) and Au(110) surfaces are larger than those for the Au(111) surface, and the friction forces for the Au(110) surface are the biggest.

**Fig. 9:**
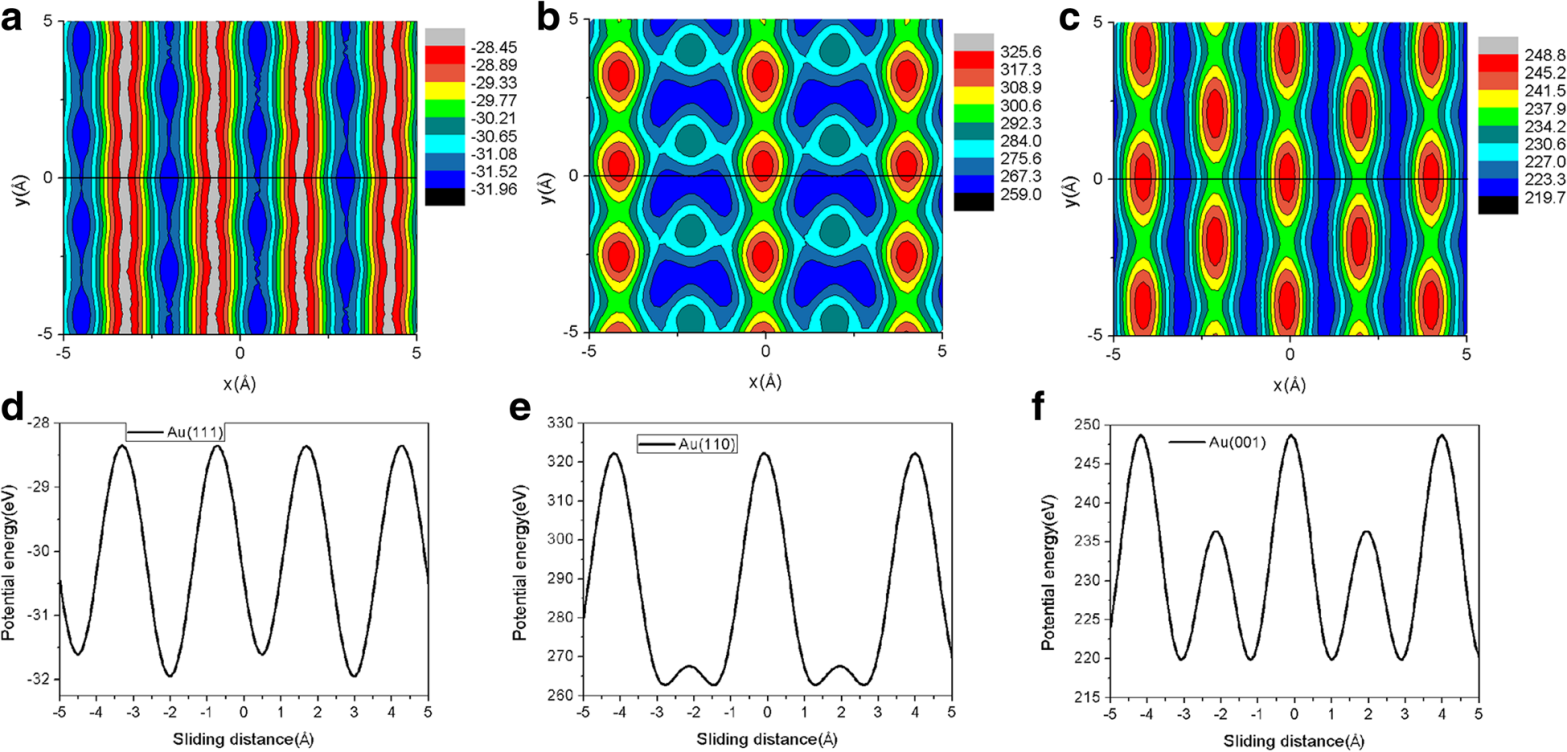
Contour maps of the potential energy for the Au(111), Au(110), and Au(001) surface at *L* = 120 nN are shown in **a**–**c**, respectively. The 5.8-nm square graphene flake is adopted. In **a**–**c**, a black solid line (*y* = 0) on the maps is used to show the sliding path of the flake. The graphene-gold interaction potential energy along the sliding path for the Au(111), Au(110), and Au(001) surface is also plotted in **d**–**f**, respectively. The unit of the potential energy is eV. The average height of the flake at *L* = 120 nN for the Au(111), Au(110), and Au(001) surface is 2.36 Å, 2.1 Å, and 2.17 Å, respectively

According to the well-known Prandtl-Tomlinson model [44], friction force is closely related to the interfacial energy corrugation [45–47]. To explore the underlying mechanisms behind the significant anisotropic effect of friction, we calculated the interaction potential energy between the flake and Au substrate as we changed the flake position [46]. The potential energy is calculated for a rigid flake at a fixed height that corresponds to the average height for the given load [29]. Three typical contour plots representing the spatial variation of the potential energy for Au(111), Au(110), and Au(001) surfaces at *L* = 120 nN are shown in Fig. 9a–c, respectively. To obtain the potential energy surface (PES) maps in Fig. 9, we use 21 mesh points along both the *x* and *y* directions. In Fig. 9, the energy corrugations calculated for Au(111), Au(110), and Au(001) are 3.5 eV, 66.6 eV, and 29.1 eV, respectively. In Fig. 9a–c, a black solid line (*y* = 0) on the PES maps is used to show the sliding path of the flake. The graphene-gold interaction potential energy along the sliding path for Au(111), Au(110), and Au(001) is also plotted in Fig. 9d–f, respectively. The energy corrugations along the sliding path for Au(111), Au(110), and Au(001) in Fig. 9 are 3.5 eV, 59.7 eV, and 29.1 eV, respectively. It can be clearly seen that the amplitude of the energy corrugation shows the same anisotropic effect as friction. The energy corrugation for the Au(001) and Au(110) surfaces is larger than that for the Au(111) surface, and the energy corrugation for the Au(110) surface is the biggest. Therefore, this clearly explains the significant anisotropic effect of friction during the sliding process [45–47]. The finding that the friction force decreases with the decrease of graphene-substrate interaction strength (energy corrugation) is consistent with the MD simulations [16] and QCM experiment [17].

**Fig. 10:**
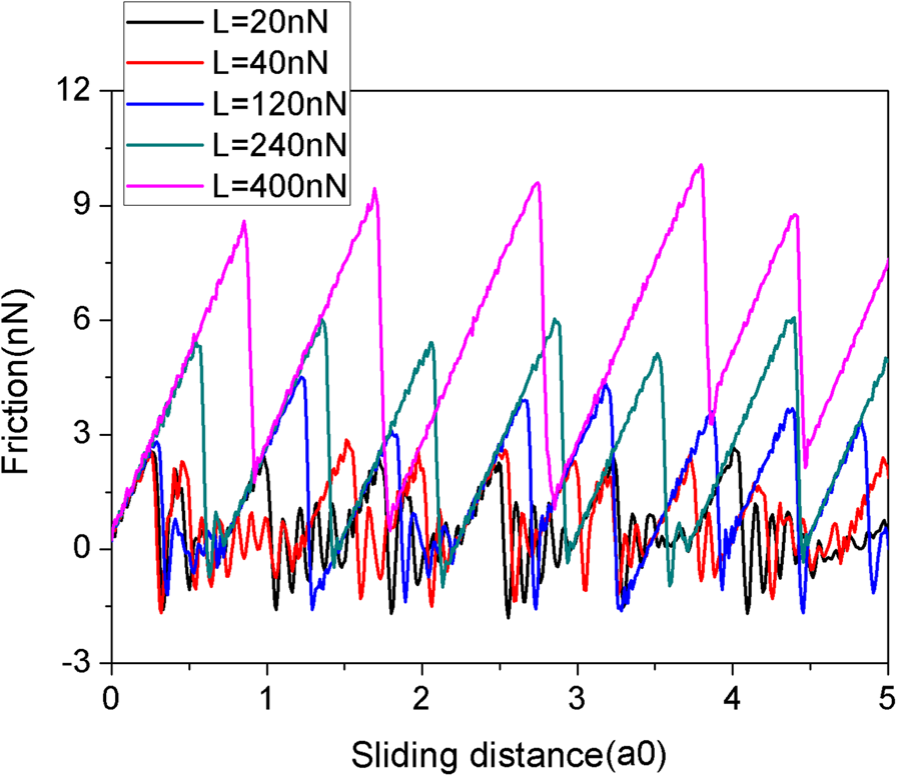
Friction force as a function of sliding distance at various normal loads (*L*) for the friction process without the movement constraint of graphene in the *y* direction. The flake has a square shape with a size of 5.8 nm. Here, a0 (= 9.99 Å) is the lattice spacing of Au(111) along the sliding direction

**Fig. 11:**
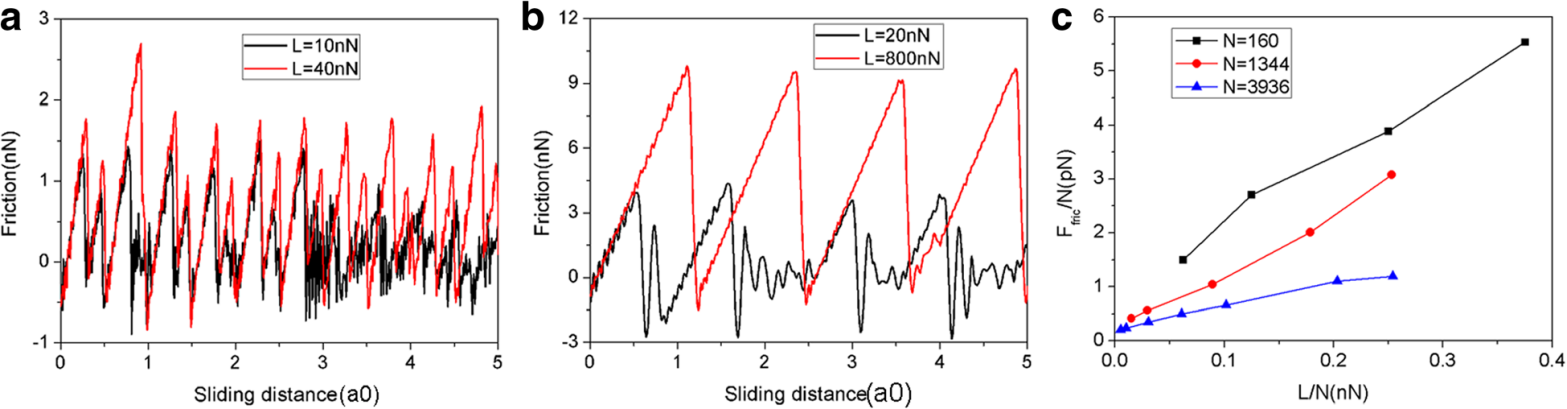
The variation of friction force and average friction force for different flake sizes for the friction process without the movement constraint of graphene in the *y* direction. The typical friction force as a function of sliding distance for the 2.0 nm (*N* = 160 atoms) flake (**a**) and 10 nm (*N* = 3936 atoms) flake (**b**). **c** The average friction force per atom (*F*_fric_/N) as a function of load per atom (L/N). Here, a0 (= 9.99 Å) is the lattice spacing of Au(111) along the sliding direction

In order to better match the real experimental conditions, we further performed MD simulations of sliding friction without the movement constraint of graphene in the *y* direction, in which case the flake can rotate and move in the *y* direction. Figure 10 shows the friction force as a function of sliding distance at various normal loads for the graphene flake with a square shape and size of 5.8 nm consisting of 1344 atoms. Although the values of friction force have changed, it can be seen that the friction forces experience continuous increase followed by abrupt drops, which is an obvious stick-slip motion similar to Fig. 2. The friction force increases with the load as expected. We also studied the friction process of another two square graphene flakes with sizes of 2.0 nm (*N* = 160 atoms) and 10.0 nm (*N* = 3936 atoms) without the movement constraint of graphene in the *y* direction. The variation of friction force and average friction force for different flake sizes during the sliding process are shown in Fig. 11. Similar to Fig. 3, we also observe an obvious stick-slip friction for both the 2.0- and 10-nm flakes. Furthermore, there exists a size effect in the average friction force per atom *F*_fric_ /N, see Fig. 11c. Under the same loads, the average friction forces per atom *F*_fric_/N are bigger for a smaller flake, which is a typical of size effect of friction. Overall, we found that the main findings of MD simulations of friction process with *y*-direction motion of flake constrained still holds after relaxing the motion constraint of graphene flake in the *y* direction during sliding.

## Conclusions

In this work, molecular dynamics simulations are employed to investigate the sliding friction behaviors of mobile graphene flakes over a single crystalline gold substrate. The effects of flake size, flake shape, relative rotation angle, and crystal orientation of substrate are thoroughly studied. It is found that there exists a size effect in the friction behaviors. Under the same load, the average friction forces per atom *F*_fric_/N are bigger for a smaller graphene flake. It is also found that flake shape plays a significant role in the friction process. The average friction forces per atom *F*_fric_/N for the square flake are much bigger than those for the triangular and round flakes. Moreover, the average friction forces per atom *F*_fric_/N for the triangular flake are the smallest. We also found that the effect of orientation of graphene flake relative to Au substrate is critical in determining friction. The friction forces for the graphene flake sliding along the armchair direction are much bigger than those for the flakes with rotation. The super low friction forces can be observed for flakes with θ = 15° and θ = 45°. Furthermore, the friction force is bigger for a larger rotation angle for the flakes with θ = 30°, 60°, and θ = 90°. In addition, it is found that the friction exhibits a significant anisotropic effect. The friction forces for the Au(001) and Au(110) surfaces are larger than those for the Au(111) surface, and the friction forces for the Au(110) surface are the biggest. This anisotropic effect of friction is attributed to the anisotropic effect of potential energy corrugation. These results not only provide insights into the underlying mechanisms of graphene flake sliding on gold substrate but also may guide the design and fabrication of nanoscale graphene-based devices.
